# Comparison of the Efficacy of Melasma Treatments: A Network Meta-Analysis of Randomized Controlled Trials

**DOI:** 10.3389/fmed.2021.713554

**Published:** 2021-09-29

**Authors:** Yi Liu, Shanshan Wu, Haixuan Wu, Xuelei Liang, Dechao Guo, Fenglin Zhuo

**Affiliations:** ^1^Department of Dermatology, Beijing Friendship Hospital, Capital Medical University, Beijing, China; ^2^National Clinical Research Center of Digestive Diseases, Beijing Friendship Hospital, Capital Medical University, Beijing, China; ^3^Department of General Surgery, The Third Affiliated Hospital of Zhejiang Chinese Medical University, Hangzhou, China

**Keywords:** melasma, efficacy, treatment, comparison, network meta-analysis

## Abstract

**Background:** Melasma is an acquired pigmentation disorder with challenges in treatment because of its refractory nature and high risk of recurrence.

**Objectives:** This study aimed to compare the efficacy and side effects of 14 common therapies for melasma using a systematic review and network meta-analysis (NMA).

**Methods:** The PubMed, Embase, and Cochrane Library databases were searched till December 2020 using the melasma area and severity index as a therapeutic index. A total of 59 randomized controlled trials (RCTs) met the inclusion criteria and were selected.

**Results:** The ranking of relative efficacy compared with placebo in descending order was Q-switched Nd:Yag 1,064-nm laser (QSND), intense pulsed light, ablative fractional laser (AFL), triple combined cream (TCC), topical vitamin C, oral tranexamic acid (oTA), peeling, azelaic acid, microneedles (MNs), topical tranexamic acid (tTA), tretinoin, picosecond laser, hydroquinone (HQ), and non-AFL. Moreover, QSND was more effective than HQ and tTA against melasma. The ranking of percentage (%) of side effects in ascending order for each of 14 therapies with more than 80 participants was tretinoin (10.1%), oTA (17.6%), HQ (18.2%), AFL (20.0%), QSND (21.5%), TCC (25.7%), tTA (36.75%), peeling (38.0%), and MN (52.3%). Taking both efficacy and safety into consideration, TCC was found to be the most favorable selection among the topical drugs for melasma. QSND and AFL were still the best ways to treat melasma among photoelectric devices. oTA as system administration was a promising way recommended for melasma. Among 31 studies, 87% (27/31) studies showed that the efficacy of combination therapies is superior to that of single therapy. The quality of evidence in this study was generally high because of nearly 50% of split-face RCTs.

**Conclusions:** Based on the published studies, this NMA indicated that QSND, AFL, TCC, and oTA would be the preferred ways to treat melasma for dermatologists. However, more attention should be paid to the efficacy and safety simultaneously during the clinical application. Most of the results were in line with those of the previous studies, but a large number of RCTs should be included for validation or update.

**Systematic Review Registration:** identifier: CRD42021239203.

## Introduction

Melasma is a common and acquired skin disorder with pigmentation that predominantly affects women with darker skin types, such as Fitzpatrick skin types III and IV (1, 2). Melasma presents with bilateral, irregularly shaped, dark brown macules and usually appears on the cheeks, forehead, nose, or upper lip on the face (3, 4). The etiology of melasma is complicated; multiple factors, such as genetics, UV rays, pregnancy, and the use of hormonal contraceptives, maybe involved (5).

The treatment of melasma has been challenging because of its unclear etiology, stubbornly refractory nature, and frequent relapse. Besides, hyperpigmentation after treatment and long-term treatment makes it more difficult (6, 7). At present, the therapies of melasma can generally be divided into two categories: nonenergy-dependent and energy-dependent therapies. The former contains topical medicine such as hydroquinone (HQ), triple combined cream (TCC), azelaic acid (AA), tranexamic acid (TA), visible and UV light protection cream, chemical peelings, microneedles (MNs), platelet-rich plasma (PRP), ultrasound therapy, and so forth (8, 9). Nonenergy-dependent therapies, which have few risks of hyperpigmentation, take effect slowly. Energy-dependent therapies include intense pulsed light (IPL), Q-switched (QS) laser, picosecond laser (PICO), fractional laser, and so forth (10). Dark brown macules in patients with melasma were found to disappear quickly with energy-dependent therapies but easily relapsed or aggravated. Based on the literature review of the evidence, the first-line therapy was topical therapy, such as HQ cream and TCC, for at least 3 months. The second-line treatment indicated combination therapy with the first-line treatment and chemical peelings. The third-line therapy was the addition of laser and light-based devices to the aforementioned treatments (11). No single treatment that is particularly effective is available. Currently, combination therapies are preferable. Park et al. found that Q-switched Nd:Yag 1,064-nm laser (QSND) combined with glycolic acid (GA) had a beneficial effect on recalcitrant mixed-type melasma, with minimal side effects (12). Nouri et al. showed slightly higher efficacy of CO_2_ laser + QS alexandrite laser than CO_2_ laser alone (13).

Selecting the best combination therapies from a variety of treatments is worth exploring. In recent years, a larger number of randomized controlled trials (RCTs) were reported for melasma, but they were limited to comparisons between two or three treatment methods. Quantitative comparisons of the efficacy between energy-dependent and nonenergy-dependent therapies for melasma would be a valuable tool for dermatologists, significantly guiding treatment decisions and influencing patient outcomes. Although multiple treatments are available for melasma, no evidence-based ranking exists for it. In this study, the first network meta-analysis (NMA) of melasma treatments was conducted using 59 selected RCTs including 14 common interventions: nonenergy-dependent treatments (HQ, topical tranexamic acid (tTA), oral tranexamic acid (oTA), AA, TCC, peeling, topical vitamin C (tVC), tretinoin, and MN) and energy-dependent treatments [IPL, QSND, PICO, ablative fractional laser (AFL), and nonablative fractional laser (NAFL)]. The efficacy of 14 therapies was directly and indirectly compared with placebo using the mean change of melasma area and severity index (MASI) before and after treatment as the outcome measure. This study aimed to provide an evidence-based comparison for treating melasma.

## Materials and Methods

This study is registered on the International Prospective Register of Systematic Review PROSPERO, number CRD42021239203.

### Databases and Search Strategy

The databases PubMed, Embase, and Cochrane Library were searched for original studies, and the search ended on December 2020. The keywords used were “melasma” or “chloasma” in the title/abstract, and filters for “randomized controlled trial” and “controlled clinical trial” were applied to the search.

### Study Selection

This systematic review and meta-analysis were conducted in agreement with the 2015 modified 32-item PRISMA extension statement for NMA (14). Only RCTs published in English were eligible for inclusion. RCTs could be placebo control studies or comparison trials of different therapies including single or combination therapy. Outcomes of interest were MASI and adverse events. MASI measures included evaluation of MASI, modified MASI (mMASI), or hemi-MASI. Some uncommon treatments for melasma were excluded, which included PRP, cysteamine, pulsed dye laser, and silymarin andrumex occidentalis.

### Data Extraction and Quality Assessment

The following details were extracted: trial information (author, publication year, sample size, trial duration, and types of intervention), population characteristics (age and skin types), outcomes of efficacy (change in MASI), and adverse effects (events and numbers). The time regime with the biggest change in MASI was selected as assessment endpoints. The risk of bias among the included studies was assessed according to the Cochrane risk-of-bias (ROB) tool (15).

### Data Synthesis and Analysis

#### Methods for Direct Comparisons of Treatments

DerSimonian and Laird random-effects model was used for standard paired meta-analysis. The standardized mean difference (SMD) of 95% CI of each result was calculated as the efficacy index.

#### Methods for Indirect and Mixed Comparisons

The random-effects NMA was performed within the Bayesian framework (16). The SMD of each result with 95% CI was summarized. The ranking of treatments was summarized and reported as the surface under the cumulative ranking curve (SUCRA) and mean ranks. The SUCRA is a percentage interpreted as the probability of the most effective treatment.

#### Examination of Assumptions in NMA

The mean difference (MD) was used to analyze the inconsistency between direct evidence and indirect evidence (17). A *P* < 0.05 indicated a significant inconsistency between direct evidence and indirect evidence in the network.

STATA version 15.0 software (StataCorp LLC, 4905 Lakeway Drive, college station, TX 77845, USA) was used for data analysis, namely, pairwise meta-analysis, estimation of inconsistency, and forest plot. The ROB was performed using RevMan 5.3 (The Nordic Cochrane Centre, The Cochrane Collaboration, Copenhagen, Denmark).

## Results

### Results of the Search

**Figure 1:**
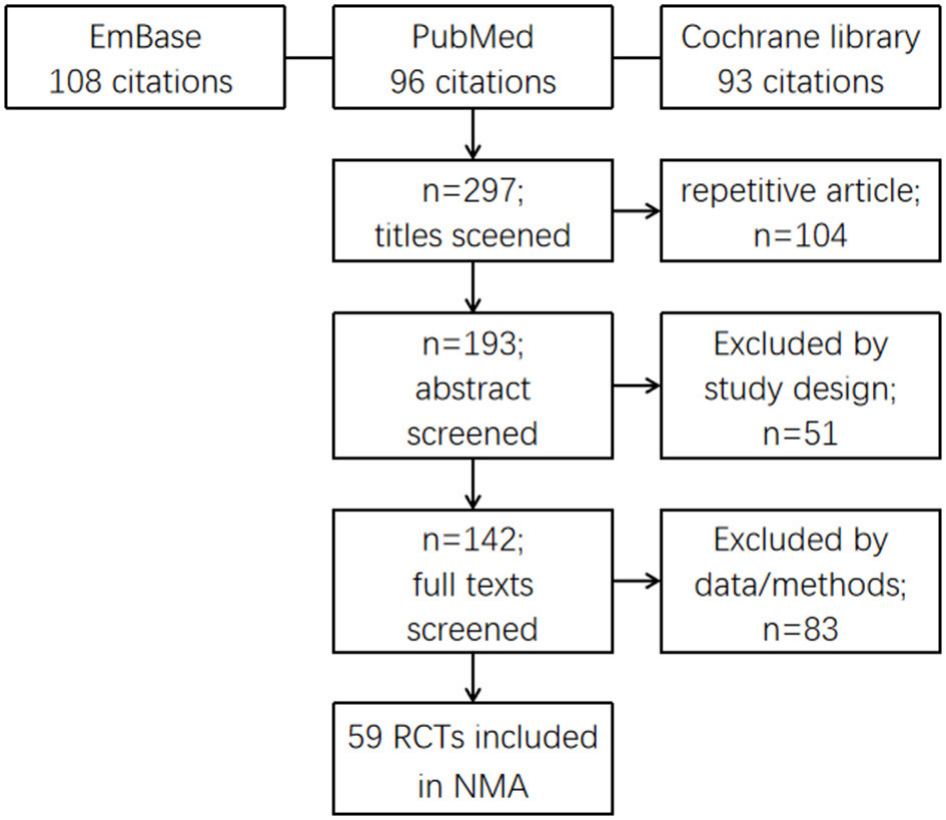
Flow chart showing the screening process of the literature.

A total of 297 studies were identified by the literature search (PubMed, Embase, and Cochrane Libraries) (Figure 1). Only 59 studies met the inclusion criteria and were included in the quantitative NMA. Detailed information for all these studies presented in the NMA is shown in Supplementary Table 1.

The included trials had 2,812 randomized participants, of whom 7.1% were men. The average age of the participants was 39.25 ± 6.69 years, and the score of MASI at baseline was 11.34 ± 4.51. A total of 40 trials among 59 included studies clearly reported Fitzpatrick skin types as follows: IV (*n* = 451), III (*n* = 353), V (*n* = 332), II (*n* = 92), and VI (*n* = 1). For the limited records of melasma type, the epidermal type (*n* = 280) was the most common type among the participants, followed by mixed type (*n* = 105) and dermal type (*n* = 57). Except for three studies that treated only one session, the average duration of treatment in the remaining studies was 12 weeks, ranging from 5 to 30 weeks. The final assessment endpoint was 4–40 weeks, with an average of 13 weeks; 26 studies involved single treatment and 33 involved combination therapy. Again, 25 of 59 studies were split-face control studies, nine studies were performed in more than two treatment arms, and the rest were pairwise comparative studies.

**Figure 2:**
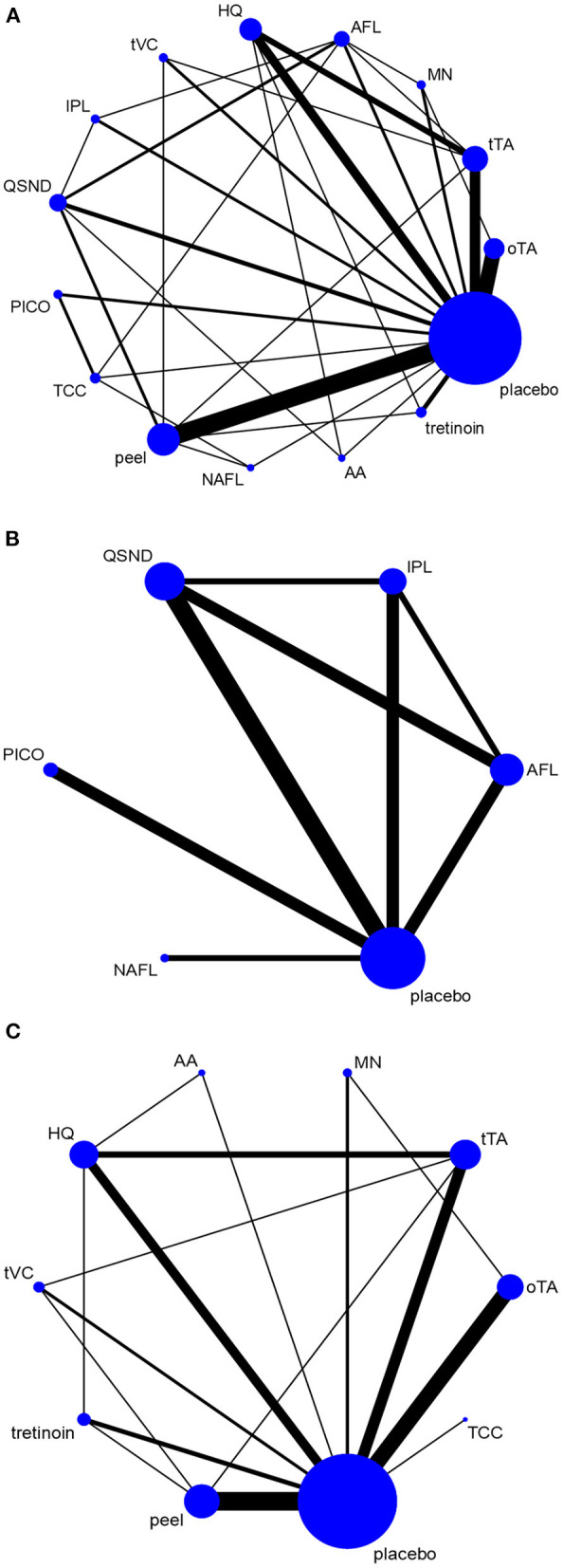
Network graph. The network graph shows the evidence network for all selected interventions. **(A)** Network graph for all selected interventions; **(B)** network graph for energy-dependent treatments; and **(C)** network graph for nonenergy-dependent treatments.

The total network graph is provided in Figure 2A. The graph included placebo and 14 treatments, of which five therapies were energy dependent and nine were nonenergy dependent. Figure 2B shows the network graph of energy-dependent treatments compared with placebo. Figure 2C presents the graph of nonenergy-dependent treatments compared with placebo.

### Risk of Bias

**Figure 3:**
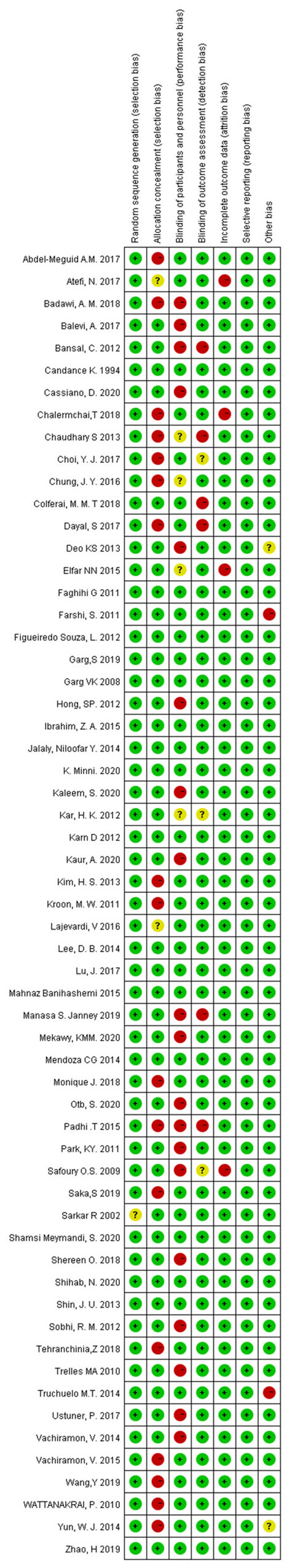
Risk of bias summary for individual studies.

**Figure 4:**
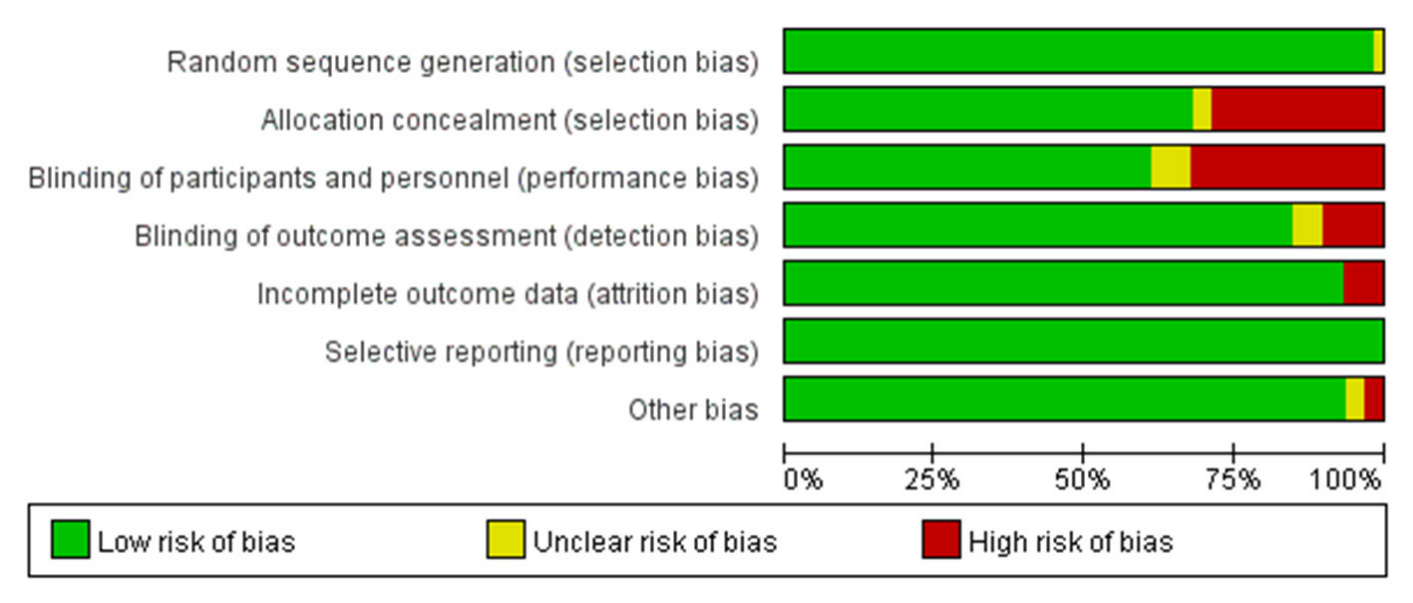
Risk of bias graph: review judgments of authors about each risk of bias item presented as percentages across all included studies.

The ROB graph evaluated using the Cochrane Collaboration's tool is shown in Figures 3, 4. Generally, low-risk RCTs account for more than 80% in six domains except for one domain (performance bias), which is attributed to 37.5% (22/59) split-face trials and 5% (3/59) without special description. The random sequence generation (selection bias) in all studies (59/59, 100%) was properly described; 94.9% (56 of 59) RCTs had a low risk of allocation concealment (selection bias), and 83.0% (49 of 59) had a low risk of detection bias. In addition, 6.7% (4 of 59) and 8.4% (5 of 59) RCTs were unclear in reporting bias and other bias, separately. Overall, the ROB was low.

### Direct Pairwise Meta-Analysis and NMA

#### Direct Pairwise Meta-Analysis

**Figure 5:**
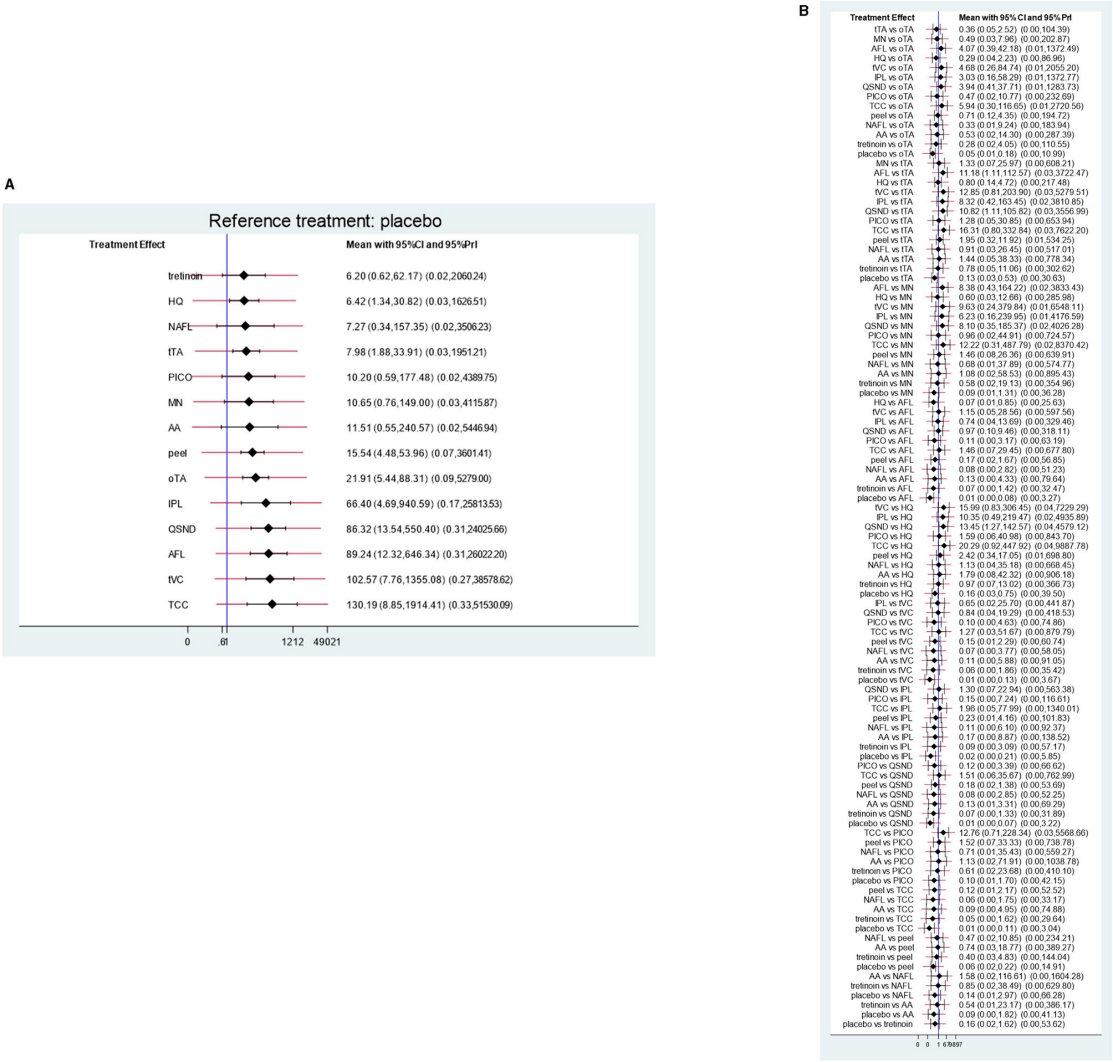
Direct comparisons. **(A)** Direct comparisons with placebo for each treatment; **(B)** direct comparisons for all treatments.

The meta-analysis of direct pairwise comparisons (Figure 5A) showed that 14 treatments exhibited greater efficacy than placebo. The most effective treatment was TCC (MD [95% CI]: 130.19 [8.85, 1,914.41]), followed by tVC (MD [95% CI]: 102.57 [7.76, 1,355.08]), AFL (MD [95% CI]: 89.24 [12.32, 646.34]), and QSND (MD [95% CI]: 86.32 [13.54, 550.40]); tretinoin (MD [95% CI]: 6.20 [0.62, 62.17]) was the worst. Direct comparisons for all treatments are shown in Figure 5B.

#### Mixed Treatment Comparisons

**Figure 6:**
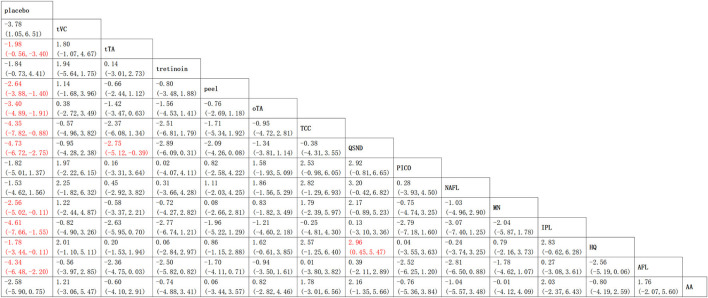
Relative effects table. Comparison of the included interventions: mean difference (95% credible intervals). Each cell gives the effect of the column-defining intervention relative to the row-defining intervention.

The results of the NMA comprising monotherapies and combination treatments are shown in Figure 6. It indicates that 64.3% of treatments (9 of 14) in the relative effects were superior to placebo. Additionally, QSND demonstrated greater efficacy over tTA and HQ (2.75 [5.12, 0.39] and 16.615 [0.45, 5.47]).

### Inconsistency Analysis

**Figure 7:**
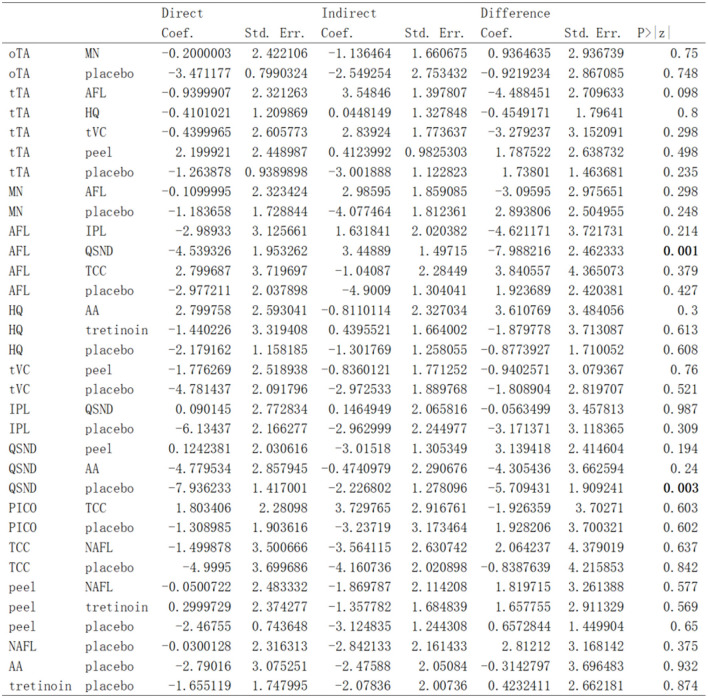
Results from the node-splitting analysis of consistency/inconsistency comparisons.

The results from the node-splitting analysis of consistency/inconsistency comparisons are shown in Figure 7. The *P* value was used to measure consistency by calculating the probability of observing the results from samples of data, assuming that the null hypothesis was true. The smaller the *P* value, the greater the inconsistency. A *P* < 0.05 indicated significant inconsistency between direct and indirect evidence in the network. In general, no significant difference was found (total *P* = 0.181) in four out of the 33 *P* value measures; only two comparisons indicated significant differences.

### Ranking of Treatments by Efficacy

The SUCRA was used as a probability to evaluate the efficacy of the treatment. SUCRA is a percentage representing the efficacy of each intervention compared with the placebo. It is used to provide the hierarchy of treatments and illustrate the location and variance of all relevant therapeutic effects. The higher the SUCRA score, the higher the probability of effectiveness.

The top six of SUCRA scores demonstrated QSND with the highest SUCRA (85.0%), followed by IPL (79.6%), AFL (78.6%), TCC (75.8%), tVC (68.5%), and oTA (63.3%). HQ and NAFL reported lower scores (29.4 and 28.1%, respectively) (Table 1).

**Table 1:** Ranking of competing treatments included in the network for melisma.

**Treatment**	SUCRA^*^	**PrBest**	**MeanRank**
QSND	85.0	19.7	3.1
IPL	79.6	26.4	3.9
AFL	78.6	11.4	4.0
TCC	75.8	24.3	4.4
tVC	68.5	10.8	5.4
oTA	63.3	1.3	6.1
Peel	47.1	0.0	8.4
AA	46.3	3.5	8.5
MN	45.6	1.1	8.6
tTA	33.2	0.0	10.4
Tretinoin	32.9	0.4	10.4
PICO	32.8	0.5	10.4
HQ	29.4	0.0	10.9
NAFL	28.1	0.5	11.1
Placebo	3.6	0.0	14.5

* *SUCRA: Surface under the cumulative ranking curve. The higher the SUCRA score, the better the treatment; PrBest indicates the possibility that the treatment is the best treatment*.

*AA, azelaic acid; AFL, ablative fractional laser; HQ, hydroquinone; IPL, intense pulsed light; MN, microneedling; NAFL, nonablative fractional laser; oTA, oral tranexamic acid; PICO, picosecond laser; QSND, Q-switched Nd:Yag 1,064-nm laser; TCC, triple combination cream; tTA, topical tranexamic acid; tVC, topical vitamin C*.

### Side Effects

The summary of side effects for each monotherapy included in the NMA is listed in Table 2. The adverse effects involved burning or stinging, erythema, scaling, swelling, hyperpigmentation, edema, and so forth. Erythema and burning were the most common side effects. In this study, the participant numbers for NAFL (27), PICO (20), AA (64), tVC (31), and IPL (47) were too less to be statistically analyzed. Given each treatment with more than 80 participants was considered significant, the ranking of side effects with percentage (*n*/*N*%) in ascending order was tretinoin (10.19%), oTA (17.65%), HQ (18.20%), AFL (20%), QSND (21.5%), TCC (25.775), tTA (36.75%), peeling (38.03%), and MN (52.33%). Owing to the limited reports of side effects in detail, the results were only for reference.

**Table 2:** Summary of side effects.

**Treatment**	***N* (total number of patients)**	***N* (number of patients with SE)**	***n*/*N*%**
IPL	47	0	0.0
***Tretinoin***	***108***	11	10.1
tVC	31	5	16.1
***oTA***	***255***	45	17.6
***HQ***	***434***	79	18.2
***AFL***	***155***	31	20.0
***QSND***	***293***	63	21.5
AA	64	15	23.4
***TCC***	***97***	25	25.7
***tTA***	***302***	111	36.7
***Peel***	***213***	81	38.0
PICO	20	9	45.0
***MN***	***86***	45	52.3
NAFL	27	18	66.6

*Only the reported side effects in monotherapy studies are listed. Monotherapies with more than 80 participants are highlighted in italic boldface. The remaining monotherapies below 80 participants are in normal font.

*AA, azelaic acid; AFL, ablative fractional laser; HQ, hydroquinone; IPL, intense pulsed light; MN, microneedling; NAFL, nonablative fractional laser; oTA, oral tranexamic acid; PICO, picosecond laser; QSND, Q-switched Nd:Yag 1,064-nm laser; SE, side effect; TCC, triple combination cream; tTA, topical tranexamic acid; tVC, topical vitamin C*.

## Discussion

In recent years, the treatments of melasma received immense attention due to its refractory nature and high relapse risk. The NMA from 59 RCTs in this study compared the clinical efficacy of 14 common interventions for melasma with a mean change in MASI scores and percentage of side effects. The results showed that compared with placebo, local therapies (TCC, tTA, peeling, and HQ), systemic medication (oTA), and device-based treatments (QSND, IPL, AFL, and NAFL) demonstrated significantly greater odds ratios of MASI change. In addition, QSND showed significantly greater odds ratios of MASI decrease in comparison with tTA and HQ. Generally, the quality of evidence graded mostly moderate to high. Although two groups of direct and indirect comparison results existed, which showed inconsistency (*P* < 0.05), no inconsistency was observed on the whole (*P* = 0.1814).

The SUCRA ranking provided a summary of efficacy with 14 interventions that clinicians could refer to, but it had the potential to be misleading. Rankings need to consider the quality of evidence and the context of the main results of an NMA, which is the relative treatment effect. A treatment may rank favorably, but the low quality of evidence with the risks of bias, inconsistency, and/or imprecision may cast doubt on confidence in such a favorable ranking (18).

**Figure 8:**
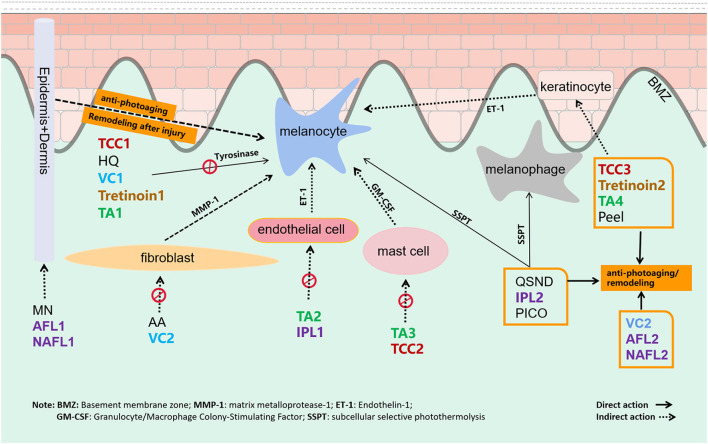
The target and mechanism of 14 common interventions for melasma are involved in this study.

In recent years, energy-based devices are becoming popular treatments for melasma. Melanocytes and removal of dermal melanophages could be destroyed through subcellular selective photothermolysis (Figure 8) (19–21). In addition, AFL and NAFL could remodel the skin tissue to reverse antiphotoaging by creating micro-areas of thermal injury. However, the risk of melanocyte irritation and postinflammatory hyperpigmentation (PIH) cannot be ignored, especially the high risk of PIH after NAFL and AFL treatment, but this also increases the risk of side effects (22, 23). The laser treatment for melasma requires the optoelectronic theory, practical ability, and experience of the operating doctor, which should be carefully selected according to the specific conditions of the doctor and the patient. According to our results, energy-based devices have different effects on melasma.

The top three treatments of the SUCRA ranking in descending order were QSND, IPL, and AFL, which were all energy-dependent therapies. Definitely, large-spot and low-energy QSND has been widely used to treat melasma, and the curative effect is obvious (24–26). Abdel et al. confirmed the effectiveness of QSND for melasma by observing the changes in dermatoscopy and *in vivo* confocal microscopy (27, 28). IPL ranked second with only three studies, and IPL-based combination therapy was discussed in two studies. In a meta-analysis-based assessment, IPL-based combination therapies had a significantly lower MASI compared with the single-treatment group (SMD = 0.61, CI [0.42, 0.80] *P* < 0.0001) (29). This result was consistent with the current ranking.

Ablative fractional laser applied in melasma included fractional CO_2_ laser and 2,940-nm Er:YAG laser. A single treatment with either fractional CO_2_ laser or 2,940-nm Er:YAG laser in two sessions with a 1-month interval reduced MASI by 48 and 26%, respectively (23, 30). AFL combined with tTA or TCC also demonstrated higher efficacy on melasma (31, 32). PICOs, as a newly emerging treatment for melasma with only three published RCTs, ranked far behind QSND, IPL, and AFL in this study. This conclusion was inconsistent with a split-face study in which 755-nm PICO was more effective than QSND for melasma in Asians (33). Another study further proved that a significantly greater decrease in melanin-induced reflectance in the spinous layer and the basal layer was observed after 755 nm of PICO treatment compared with the QSND treatment for melasma (34). More RCTs with PICO for melasma should be included in the future.

Following QSND, IPL, and AFL, TCC ranked fourth in superiority to other nonenergy-dependent treatments, such as oTA, peeling, MN, and other topical agents, which should be inseparable from the combined efficacy of three pharmaceutical ingredients (Figure 8) (35). TCC consists of HQ, a retinoid, and a fluorinated corticosteroid and is widely regarded as the safest and effective treatment for melasma (36). In a large, multicenter RCT, TCC was found to be more effective than any dual combination of the three active ingredients (37). Studies showed that the efficacy of TCC in treating melasma was far better than that of HQ (38, 39) and was equivalent to a 755-nm PICO laser (40). No direct evidence is available to compare TCC with other topical drugs. Except for TCC, the ranking of other topical agents in descending order was tVC, AA, tTA, tretinoin, and HQ. Topical vitamin C (VC) and tTA rankings were inferior to HQ in this study, which might be relevant to the delivery mode with VC iontophoresis or intradermal TA (41). Besides that, the action of multiple targets in the therapeutic mechanism is also an important factor. VC can not only inhibit the transformation of tyrosine to pigment but also be antioxidant-promoting collagen synthesis (42). Antiphotoaging in the melasma area is recently considered to be an important strategy for melasma therapy. Energy-based devices, MN, peeling, TCC, etc. are also considered to remodel the aging microenvironment of melasma (Figure 8).

Tranexamic acid also showed superior effects on melasma in this study. TA for melasma targets melanocyte, endothelial cell, mast cell, and keratinocyte to remove pigmentation (Figure 8) (43, 44). Owing to the expected effect, oTA has been recommended as first-line therapy in a recent review (11). Recently, the interest has grown in studying the effects of TA in various formulations, namely, topical, intradermal, and oral, for melasma. In a meta-analysis, oTA demonstrated the greatest improvement compared with topical and intradermal formulations (45). However, more studies are needed to determine its long-term safety and efficacy. Apart from oTA, another oral agent on melasma, *Polypodium leucotomos* extract (PLE) is an antioxidant with photoprotective effect and immunoprotective activities UVA and UVB radiation. Till now, two RCT studies of PLE on melasma have been published. At the end of the two studies, there were no significant differences between oral PLE and placebo (46, 47). This conclusion should attribute to the single target to UV radiation protectant as an adjunct to topical sunscreen.

Chemical peeling is the recommended second-line treatment for melasma, and it can accelerate epidermal renewal and collagen regeneration to remove epidermal melanin and suspend the transfer of melanosomes (48). A systematic review and meta-analysis showed that GA peeling had no obvious advantage over tretinoin (MD 0.00, 95% CI [0.99, 0.99], *P* = 0.3) and VC (MD 1.50, 95% CI [0.50, 3.50], *P* = 0.14), while TCA peeling (MD 5.30, 95% CI [6.41, 173 4.19], *P* < 0.001) and Jessner solution (MD 3.20, 95% CI [5.35, 1.05], *P* = 0.004) were better than HQ in treating melasma (49). Chemical peeling in this study included GA, TCA, SA, and Jessen solution. In the SUCRA ranking, peeling held the middle position (7/14), also ranking higher than tretinoin and HQ.

The role of microneedling in melasma ranked ninth in this study. MN creates minor skin damage, and in the process of skin self-repairment, it may reverse some structural patterns of melasma (50). Most studies assessed the use of MN in combination with other topical agents for the facilitation of drug delivery. Very few studies are available on MN monotherapy. Cassiano et al. (50) showed that a one-time treatment with dermaroller microneedling reduced mMASI by 9% after a 1-week follow-up, which was significantly more than that in the control group. Above all, the advantage of MN includes the very low risk of PIH. For the choice of melasma treatment, besides effectiveness, side effects are also an important factor. The side effects involved in published studies for 14 therapies were summarized in this study. Generally speaking, oTA, with the fifth position in relative efficacy and second position in side effects (17.6%), is a promising and preferred way for melasma therapy. This conclusion was following other studies (45, 51). Photoelectric devices, such as QSND and AFL, ranked first and third in efficacy, show similar side effects (21.5 and 20%) but a little higher than those of oTA (17.65%). In consequence, QSND and AFL are worthy of being applied as a monotherapy or combination therapy for melasma. Among topical medicines, single tretinoin or HQ is not recommended as the first choice owing to their low efficacy. We recommend HQ combined with photoelectric devices for melasma. TCC, ranking fourth in efficacy, is a favorable selection with moderate side effects (22.7%) for melasma. Peeling and MN with a high incidence of side effects in this study were not the preferable methods so far. Therefore, besides ensuring the effectiveness of treatment, the side effects after treatment should also be considered and appropriate posttreatment care should be provided to reduce the occurrence of melasma.

Neagu et al. (52) suggested that no single treatment for melasma is generally effective. Compared with a single treatment, both double and triple treatments show good curative effects (52). Among 56 included studies in the study, 31 studies compared the efficacy of combination therapy with a single therapy, of which 27 considered that the efficacy of combination therapy was better than that of single therapy, and four considered that there was no significant difference between the two. In general, the efficacy of interventions with multitarget effect in the treatment of melasma is better than those with a single target. In addition to the inhibition of melanogenesis or destruction of melanin granule, the inhibitory effect of steroids and TA on ET-1 (endothelin 1), the improvement effect of TA on blood vessels and mast cells, and the effect of the laser by reversing the characteristics of photoaging on melasma also should be taken into account. These therapeutic targets seem to play an important role in the treatment of melasma. The effect of combination therapy with multiple targets among drugs, energy-based devices or MN, and so on would be better than that of single therapy, and it is possible to reduce the recurrence of melasma.

This study was restricted by the limited number of RCTs, particularly those related to PICO laser. Hence, it is necessary to perform more RCTs and split-face studies that can reduce the ROB to update the current results and help develop more standardized protocols. These data may direct doctors to better treat patients with melasma. In clinical application, more attention needs to be paid to the efficacy and safety of the treatment. Based on this, the combination therapy of multiple targets on mechanism may produce unexpected effects. As for the ranking of melasma monotherapy, we believe that following up the release of new trials, the network will expand, and the ranking list may change a little.

## Data Availability Statement

The original contributions presented in the study are included in the article/Supplementary Material, further inquiries can be directed to the corresponding author.

## Author Contributions

YL contributed to data collection and article writing. SW contributed to statistical methods. HW participated in the article screening. XL participated in the chart making. DG participated in the article revision, and the article topic selection and final revision were in the charge of FZ. All authors contributed to the article and approved the submitted version.

## Conflict of Interest

The authors declare that the research was conducted in the absence of any commercial or financial relationships that could be construed as a potential conflict of interest.

## Publisher's Note

All claims expressed in this article are solely those of the authors and do not necessarily represent those of their affiliated organizations, or those of the publisher, the editors and the reviewers. Any product that may be evaluated in this article, or claim that may be made by its manufacturer, is not guaranteed or endorsed by the publisher.

## References

[1] Grimes PE. Melasma. Etiologic and therapeutic considerations. Arch Dermatol. (1995) 131:1453–7. 10.1001/archderm.131.12.1453 7492140

[2] Werlinger KD Guevara IL González CM Rincón ET Caetano R Haley RW . Prevalence of self-diagnosed melasma among premenopausal Latino women in Dallas and Fort Worth, Tex. Arch Dermatol. (2007) 143:424–5. 10.1001/archderm.143.3.424 17372115

[3] Rodrigues M Pandya AG. Melasma: clinical diagnosis and management options. Australas J Dermatol. (2015) 56:151–63. 10.1111/ajd.12290 25754098

[4] Ponzio HA Favaretto AL Rivitti EA. Proposal of a quantitative method to describe melasma distribution in women. J Cosmet Dermatol. (2007) 20:103–11.

[5] Handel AC Miot LD Miot HA. Melasma: a clinical and epidemiological review. An Bras Dermatol. (2014) 89:771–82. 10.1590/abd1806-4841.20143063 25184917 PMC4155956

[6] Kwon SH Na JI Choi JY Park KC. Melasma: Updates and perspectives. Exp Dermatol. (2019) 28:704–8. 10.1111/exd.13844 30422338

[7] Sanchez NP Pathak MA Sato S Fitzpatrick TB Sanchez JL Mihm MC. Melasma: a clinical, light microscopic, ultrastructural, and immunofluorescence study. J Am Acad Dermatol. (1981) 4:698–710. 10.1016/S0190-9622(81)70071-9 6787100

[8] Sirithanabadeekul P Dannarongchai A Suwanchinda A. Platelet-rich plasma treatment for melasma: A pilot study. J Cosmet Dermatol. (2020) 19:1321–7. 10.1111/jocd.13157 31568636

[9] Austin E Nguyen JK Jagdeo J. Topical treatments for melasma: A systematic review of randomized controlled trials. J Drugs Dermatol. (2019). 18:S1545961619P1156X. 31741361

[10] Rivas S Pandya AG. Treatment of melasma with topical agents, peelings and lasers: an evidence-based review. Am J Clin Dermatol. (2013) 14:359–76. 10.1007/s40257-013-0038-4 23881551

[11] Spierings NMK. Melasma: A critical analysis of clinical trials investigating treatment modalities published in the past 10 years. J Cosmet Dermatol. (2020) 19:1284–9. 10.1111/jocd.13182 31603285

[12] Park KY Kim DH Kim HK Li K Seo SJ Hong CK. randomized, observer-blinded, comparison of combined 1064-nm Q-switched neodymium-doped yttrium–aluminum–garnet laser plus 30% glycolic acid peeling vs. laser monotherapy to treat melisma. J Clin Exp Dermatol Res. (2011) 36:864–70. 10.1111/j.1365-2230.2011.04150.x 21973194

[13] Nouri K Bowes L Chartier T Romagosa R Spencer J. Combination treatment of melasma with pulsed CO2 laser followed by Q-switched alexandrite laser: a pilot study. Dermatol Surg. (1999) 25:494–7. 10.1046/j.1524-4725.1999.08248.x 10469101

[14] Hutton B Salanti G Caldwell DM Chaimani A Schmid CH Cameron C . The PRISMA extension statement for reporting of systematic reviews incorporating network meta -analyses of health care interventions: checklist and explanations. Ann Intern Med. (2015) 162:777–84. 10.7326/M14-2385 26030634

[15] Higgins JPT Green S. Cochrane Handbook for Systematic Reviews of Interventions Version 5.1.0. (2011). Available online at: http://handbook.cochrane.org/ (accessed April 27, 2016).

[16] Salanti G. Indirect and mixed-treatment comparison, network, or multiple treatments meta-analysis: Many names, many benefits, many concerns for the next generation evidence synthesis tool. Res Synth Methods. (2012) 3:80–97. 10.1002/jrsm.1037 26062083

[17] Higgins JP Jackson D Barrett JK Lu G Ades AE White IR. Consistency and inconsistency in networkmeta-analysis: concepts and models for multi-arm studies. Res Synth Methods. (2012) 3:98–110. 10.1002/jrsm.1044 26062084 PMC4433772

[18] Mbuagbaw L Rochwerg B Jaeschke R Heels-Andsell D Alhazzani W Thabane L . Approaches to interpreting and choosing the best treatments in network meta-analyses. Syst Rev. (2017) 6:79. 10.1186/s13643-017-0473-z 28403893 PMC5389085

[19] Kim JH Kim H Park HC Kim IH. Subcellular selective photothermolysis of melanosomes in adult zebrafish skin following 1064-nm Q-switched Nd:YAG laser irradiation. J Invest Dermatol. (2010) 130:2333–5. 10.1038/jid.2010.129 20463692

[20] DiBernardo BE Pozner JN. Intense pulsed light therapy for skin rejuvenation. Clin Plast Surg. (2016) 43:535–40. 10.1016/j.cps.2016.03.008 27363767

[21] Sheth VM Pandya AG. Melasma: a comprehensive update: part II. J Am Acad Dermatol. (2011) 65:699–714. 10.1016/j.jaad.2011.06.001 21920242

[22] Manaloto RM Alster T. Erbium: YAG laser resurfacing for refractory melasma. Dermatol Surg. (1999) 25:121–3. 10.1046/j.1524-4725.1999.08103.x 10037517

[23] El-Sinbawy Zenab G Abdelnabi N M Sarhan N E Elgarhy LH. Clinical & ultrastructural evaluation of the effect of fractional CO2 laser on facial melisma. Ultrastruct Pathol. (2019) 43:135–44. 10.1080/01913123.2019.1673861 31575311

[24] Sim JH Park YL Lee JS Lee SY Choi WB Kim HJ . Treatment of melasma by low-fluence 1064 nm Q-switched Nd:YAG laser. J Dermatolog Treat. (2014) 25:212–7. 10.3109/09546634.2012.735639 23030603

[25] Kaminaka C Furukawa F Yamamoto Y. The clinical and histological effect of a low-fluence Q-switched 1,064-nm neodymium: yttrium-aluminum-garnet laser for the treatment of melasma and solar lentigenes in asians: prospective, randomized, and split-face comparative study. Dermatol Surg. (2017) 43:1120–33. 10.1097/DSS.0000000000001120 28328709

[26] Choi JE Lee DW Seo SH Ahn HH Kye YC. Low-fluence Q-switched Nd:YAG laser for the treatment of melasma in Asian patients. J Cosmet Dermatol. (2018) 17:1053–8. 10.1111/jocd.12760 30280474

[27] Abdel HR Mohammed FN Sayed KS Abd EI Fattah NA Ibrahim S. Dermoscopy as a useful tool for evaluating melasma and assessing the response to 1064-nm Q-switched Nd:YAG laser. Dermatol Ther. (2020) 33:e13629. 10.1111/dth.13629 32431000

[28] Longo C Pellacani G Tourlaki A Galimberti M Bencini PL. Melasma and low-energy Q-switched laser: treatment assessment by means of in vivo confocal microscopy. Lasers Med Sci. (2014) 29:1159–63. 10.1007/s10103-013-1498-8 24292199

[29] Yi J Hong T Zeng H Li P Li P Wang S . A meta-analysis based assessment of intense pulsed light for treatment of melasma. Aesthetic Plast Surg. (2020) 44:947–52. 10.1007/s00266-020-01637-x 32055937

[30] Wanitphakdeedecha R Manuskiatti W Siriphukpong S Chen TM. Treatment of melasma using variable square pulse Er:YAG laser resurfacing. Dermatol Surg. (2009). 35:475–81. 10.1111/j.1524-4725.2009.01066.x 19250309

[31] Badawi AM Osman MA. Fractional erbium-doped yttrium aluminum garnet laser-assisted drug delivery of hydroquinone in the treatment of melasma. Clin Cosmet Investig Dermatol. (2018). 11:13–20. 10.2147/CCID.S147413 29379308 PMC5757209

[32] Farshi S. Comparative study of therapeutic effects of 20% azelaic acid and hydroquinone 4% cream in the treatment of melasma. J Cosmet Dermatol. (2011) 0:282–7. 10.1111/j.1473-2165.2011.00580.x 22151936

[33] Lee MC Lin YF Hu S Huang YL Chang SL Cheng CY . A split-face study: comparison of picosecond alexandrite laser and Q-switched Nd:YAG laser in the treatment of melasma in Asians. Lasers Med Sci. (2018) 33:1733–8. 10.1007/s10103-018-2529-2 29732522

[34] Jo DJ Kang IH Baek JH Gwak MJ Lee SJ Shin MK. Using reflectance confocal microscopy to observe in vivo melanolysis after treatment with the picosecond alexandrite laser and Q-switched Nd:YAG laser in melasma. Lasers Surg Med. (2018) 111:40–8. 10.1002/lsm.23025 30351494

[35] Kligman AM Willis I. A new formula for depigmenting human skin. Arch Dermatol. (1975) 111:40–8. 10.1001/archderm.111.1.40 1119822

[36] McKesey J Tovar-Garza A Pandya AG. Melasma treatment: an evidence-based review. Am J Clin Dermatol. (2020) 21:173–225. 10.1007/s40257-019-00488-w 31802394

[37] Taylor SC Torok H Jones T Lowe N Rich P Tschen E . Efficacy and safety of a new triplecombination agent for the treatment of facial melasma. Cutis. (2003) 72:67–72. 10.1016/S1040-0486(03)00017-6 12889718

[38] Ferreira CT Hassun K Sittart A Lourdes VMA. comparison of triple combination cream and hydroquinone 4% cream for the treatment of moderate to severe facial melasma. J Cosmet Dermatol. (2007) 6:36–9. 10.1111/j.1473-2165.2007.00288.x 17348994

[39] Chan R Park KC Lee MH Lee ES Chang SE Leow YH . A randomized controlled trial of the efficacy and safety of a fixed triple combination (fluocinolone acetonide 001%, hydroquinone 4%, tretinoin 005%) compared with hydroquinone 4% cream in Asian patients with moderate to severe melisma. Br J Dermatol. (2008) 159:697–703. 10.1111/j.1365-2133.2008.08717.x 18616780

[40] Wang YJ Lin ET Chen YT Chiu PC Lin BS Chiang HM . Prospective randomized controlled trial comparing treatment efficacy and tolerance of picosecond alexandrite laser with a diffractive lens array and triple combination cream in female Asian patients with melasma. J Eur Acad Dermatol Venereol. (2020) 34:624–32. 10.1111/jdv.15934 31494973

[41] Saki N Darayesh M Heiran A. Comparing the efficacy of topical hydroquinone 2% versus intradermal tranexamic acid microinjections in treating melasma: a split-face controlled trial. J Dermatolog Treat. (2018) 29:405–10. 10.1080/09546634.2017.1392476 29027510

[42] Farris PK. Topical vitamin C: A useful agent for treating photoaging and other dermatologic conditions. Dermatol Surg. (2005) 31:814–7. 10.1111/j.1524-4725.2005.31725 16029672

[43] Na JI Choi SY Yang SH Choi HR Kang HY Park KC. Effect of tranexamic acid on melasma: a clinical trial with histological evaluation. J Eur Acad Dermatol Venereol. (2013) 27:1035–9. 10.1111/j.1468-3083.2012.04464.x 22329442

[44] Kim SJ Park JY Shibata T Fujiwara R Kang HY. Efficacy and possible mechanisms of topical tranexamic acid in melasma. Clin Exp Dermatol. (2016) 41:480–5. 10.1111/ced.12835 27135282

[45] Wang JV Jhawar N Saedi N. Tranexamic Acid for Melasma: Evaluating the Various Formulations. J Clin Aesthet Dermatol. (2019) 12:E73–4. 31531176 PMC6715124

[46] Chee-Leok G Sai Yee C Thng Guan TS Alejandra VM Arancha DR. Polypodium leucotomos double-blind, placebo-controlled trial to evaluate the effectiveness of extract in the treatment of melasma in asian skin: a pilot study. J Clin Aesthet Dermatol. (2018). 11:14–19. 29606995 PMC5868779

[47] Ahmed Ammar M. Lopez Isha, Perese Francisco, Vasquez Rebecca, Hynan Linda S, Chong Benjamin, et al. A randomized, double-blinded, placebo-controlled trial of oral Polypodium leucotomos extract as an adjunct to sunscreen in the treatment of melisma. JAMA Dermatol. (2013) 149:981–3. 10.1001/jamadermatol.2013.4294 23740292

[48] Sarkar R Arsiwala S Dubey N. Sonthalia Sidharth, Das Anupam, Arya Latika, et al. Chemical Peels in Melasma: A Review with Consensus Recommendations by Indian Pigmentary Expert Group. Indian J Dermatol. (2017) 62:578–84. 10.4103/ijd.IJD_490_17 29263530 PMC5724304

[49] Dorgham NA Hegazy RA Sharobim AK Dorgham DA. Efficacy and tolerability of chemical peelinging as a single agent for melasma in dark-skinned patients: A systematic review and meta-analysis of comparative trials. J Cosmet Dermatol. (2020) 19:2812–9. 10.1111/jocd.13725 32947652

[50] Cassiano DP Espósito ACC Hassun KM Lima EVA Bagatin E Miot HA. Early clinical and histological changes induced by microneedling in facial melasma: A pilot study. Indian J Dermatol Venereol Leprol. (2019) 85:638–41. 10.4103/ijdvl.IJDVL_44_19 31607716

[51] Sarma N Chakraborty S Poojary SA Rathi S Kumaran S Nirmal B . Evidence-based review, grade of recommendation, and suggested treatment recommendations for melasma. Indian Dermatol Online J. (2017) 8:406–42. 10.4103/idoj.IDOJ_187_17 29204385 PMC5707834

[52] Neagu N Conforti C Agozzino M Marangi GF Morariu SH Pellacani G . Melasma treatment: a systematic review. J Dermatolog Treat. (2021) 14:1–39. 10.1080/09546634.2021.1914313 33849384

