# Supplementary material for: Comparison of the Efficacy of Melasma Treatments: A Network Meta-Analysis of Randomized Controlled Trials
Source: Front Med (Lausanne). 2021 Sep 29;8:713554. doi: 10.3389/fmed.2021.713554 (PMC8511390; doi:10.3389/fmed.2021.713554)
Supplement: Supplementary file 1 [file Table_1.docx]

**Supplementary table 1.** Studies included in the network meta-analysis on various treatments for melasma

| Year /Author | Treatment | *N* | Age | Skin type | Duration (weeks) | Change of MASI (mean ± SD) | Adverse effects |
| --- | --- | --- | --- | --- | --- | --- | --- |
| 2020 Minni K^[53]^ | oTA + FbTc | 65 | 36 | III1, IV15, Ⅴ48, Ⅵ1 | 12 | 8.19 ± 3.90 | Erythema 11, burning 9, gastrointestinal complaints |
|  | FbTc | 65 | 38.5 | IV18, Ⅴ47 |  | 5.07 ± 3.47 | Erythema 17, burning 16 |
| **2020 Kaur A^[54]^** | **MN + tTA** | **40** | **33.15** | **IV36, Ⅴ4** | **8** | **2.84 ± 1.55** | **Erythema 7, pruritus 5, burning 1, hyperpigmentation 2, dryness 22** |
|  | **MN** |  |  |  |  | **0.80 ± 1.82** | **Erythema 9, pruritus 4, burning 2, hyperpigmentation 2, dryness 22** |
| 2020 Cassiano D^[55]^ | Placebo | 16 | ＞18 |  | 8 | 0.40 ± 4.10 | TA: nausea, abdominal pain, hair loss, blurred vision, headache 1; MN: herpes simplex 3 |
|  | oTA | 16 |  |  |  | 3.20 ± 4.63 |  |
|  | MN | 16 |  |  |  | 3.00 ± 3.67 |  |
|  | oTA + MN | 16 |  |  |  | 2.80 ± 7.18 |  |
| 2020 Shihab N^[56]^ | oTA + HQ | 25 | 46.6 ± 5.5 |  | 12 | 4.93 ± 2.14 | HQ: erythema, pruritus 3; TA: changes in the menstrual cycle 1 |
|  | HQ | 25 | 46.4 ± 5.8 |  |  | 0.89 ± 2.01 |  |
| **2020 Kaleem S^[57]^** | **tTA** | **60** | **36 ± 7.9** | **III34, IV24, Ⅴ2** | **12** | **1.67 ± 2.22** | **Erythema 6, swelling 31, burning 8, irritation 15** |
|  | **Placebo** |  |  |  |  | **0.01 ± 2.65** |  |
| **2020 Otb S^[58]^** | tTA | 27 | 44 ± 6.3 | Ⅱ2, Ⅲ18, IV7 | 24 | 1.89 ± 2.15 | No |
|  | AFL-Er:YAG |  |  |  |  | 0.95 ± 2.44 | No |
| 2020, Shamsi MS^[59]^ | tTA | 30 | 26.1 ± 0.6 | Ⅱ1, Ⅲ8, IV12, Ⅴ9 | 12 | 6.05 ± 4.79 | Erythema 25, PIH 2 |
|  | HQ | 30 |  | Ⅱ2, III6, IV14, Ⅴ8 |  | 6.40 ± 4.65 | Erythema 7, PIH 4 |
| **2020 Mekawy KMM^[60]^** | **MN** | **30** | **41.9 ± 7.8** | **Ⅱ5, Ⅲ23, IV2** | **14** | **1.84 ± 1.69** | **PIH 3** |
|  | **AFL-CO_2_** |  |  |  |  | **1.73 ± 1.69** |  |
| 2019 Garg S^[61]^ | IPL | 20 | 22–46 | III33, IV27 | 24 | 4.59 ± 5.20 | Dryness |
|  | QSND | 20 |  |  |  | 4.68 ± 4.55 | PIH 3, acne breakouts 2 |
|  | AFL-Er:YAG | 20 |  |  |  | 7.58 ± 8.12 | PIH 2, herpes labialis 1 |
| 2019 Wang YJ^[39]^ | PICO (3 sessions) | 9 | 40.4 |  | 20 | 4.20 ± 4.43 | Erythema 2, focal desquamation 1 |
|  | PICO (5 sessions) | 11 | 46.4 |  |  | 4.00 ± 3.93 | Erythema 3, PIH 2, focal desquamation 1 |
|  | TCC | 6 | 50 |  |  | 5.90 ± 4.49 | Dryness, erythema, and itching 2 |
| **2019 Saka S^[62]^** | **tTA** | **38** | **40.16 ± 5.76** | **Epidermal 24; dermal 5; mixed 9** | **12** | **1.11 ± 1.66** | **Erythema and burning** |
|  | **Placebo** |  |  |  |  | **0.19 ± 1.65** |  |
| 2019 Janney MS^[63]^ | tTA | 50 | 35.86 ± 7.51 | Ⅲ2, Ⅳ26, Ⅴ22 | 12 | 2.92 ± 3.10 |  |
|  | HQ | 50 | 36.32 ± 7.65 | Ⅲ2, Ⅳ24, Ⅴ24 |  | 2.39 ± 3.51 |  |
| **2019 Zhao H^[64]^** | **tTA** | **17** | **39.47 ± 6.05** | **III13, IV4** | **8** | **4.06 ± 3.38** | **Erythema 2, stuffy 1** |
|  | **tVC** |  |  |  |  | **3.62 ± 3.88** |  |
| **2018 Tehranchinia Z^[65]^** | **tTA + HQ** | **55** | **35.93 ± 5.9** | **Ⅱ9, Ⅲ35, Ⅳ11** | **16** | **3.39 ± 1.62** | **Erythema 26, pruritus 6** |
|  | **HQ** |  |  |  |  | **2.27 ± 1.69** | **Erythema 28, pruritus 7** |
| **2018 Chalermchai T^[66]^** | **PICO + HQ** | **30** | **47.5 ± 6.8** | **Ⅲ5, IV25** | **12** | **5.94 ± 2.95** | **Erythema 2, skin desquamation 2, burning 1** |
|  | **HQ** |  |  |  |  | **5.30 ± 2.95** |  |
| 2018 Colferai MMT^[67]^ | oTA | 20 | 45.75 |  | 12 | 10.10 ± 7.88 | Gastrointestinal tract 7 |
|  | Placebo | 17 | 42.88 |  |  | 1.70 ± 6.89 | No |
| **2018 Badawi AM^[31]^** | **AFL-Er:YAG + HQ** | **30** | **37.87 ± 7.97** | **Ⅲ11, IV12, V7** | **10** | **5.57 ± 2.85** | **Erythema, burning, itching** |
|  | **HQ** |  |  |  |  | **2.28 ± 2.84** | **Erythema 5, burning, itching** |
| 2018 Vanaman Wilson MJ^[68]^ | Diode laser + HQ | 20 | 46 ± 10.9 | Ⅲ14, Ⅳ4, Ⅴ2 | 12 | 7.40 ± 6.46 |  |
|  | Diode laser + placebo | 20 | 47.2 ± 8.5 | Ⅲ8, Ⅳ10, Ⅴ4 |  | 9.70 ± 9.22 |  |
| 2018 Tawfic SO^[69]^ | AFL-CO_2_ | 28 | 39.61 ± 6.71 | Ⅲ14, IV–Ⅴ14 | 18-26 | 1.75 ± 4.38 | Burning 28 |
|  | AFL-CO_2_+tTA | 13 | 40.45 ± 8.23 | Ⅲ6, IV–Ⅴ7 |  | 1.69 ± 4.12 | Burning 13, PIH 2 |
|  | AFL-CO_2_+tTA | 15 | 38.80 ± 5.21 | Ⅲ8, IV–Ⅴ7 |  | 1.45 ± 4.53 | Burning 15 |
| 2017 Atefi N^[70]^ | tTA | 30 | 38.10 ± 6.27 |  | 12 | 2.47 ± 0.93 |  |
|  | HQ | 30 | 39.97 ± 7.86 |  |  | 2.07 ± 0.82 |  |
| **2017 Choi YJ^[71]^** | **PICO + HQ** | **39** | **48.38 ± 6.65** | **Ⅲ22, IV17** | **14** | **3.70 ± 8.90** | **Dermatitis 2** |
|  | **HQ** |  |  |  |  | **1.30 ± 9.34** |  |
| 2017 Dayal S^[72]^ | GA peel + AA | 30 | 33.40 ± 7.44 | IV16, V14 | 24 | 9.33 ± 4.07 | Erythema 4, PIH 4, pruritus 2, scaling 2, burning 5 |
|  | AA | 30 | 33.77 ± 6.11 | IV16, V14 |  | 6.56 ± 4.30 | Erythema 1, PIH 2, pruritus 5, scaling 1, burning 3 |
| 2017 Lu J^[73]^ | oTA | 41 | 43 | Ⅲ or IV | 8 | 5.26 ± 3.16 | No |
|  | Placebo | 41 |  |  |  | -0.73 ± 3.16 | No |
| **2017 Abdel-Meguid AM^[74]^** | **Jessner peel + TCA peel** | **24** | **34.50 ± 7.76** | **Epidermal 14; mixed 10** | **12** | **6.23 ± 4.97** | **Erythema 24, discomfort 15, burning 14, itching 3, crustation 24, PIH 2** |
|  | **TCA peel** |  |  |  |  | **4.54 ± 5.18** | **Erythema 24, discomfort 3, burning 4, itching 2, crustation 24** |
| 2017 Balevi A^[75]^ | tVC + SA peel | 23 | 36.32 ± 10.19 |  | 8 | 11.36 ± 10.48 |  |
|  | SA peel | 21 |  |  |  | 1.84 ± 10.68 |  |
| **2017 Ustuner P^[76]^** | **QSND + tVC** | **48** | **37.69 ± 8.24** | **Ⅱ30, Ⅲ18** | **16** | **4.55 ± 3.94** | **Irritation 3, hyperpigmentation 3, hyperpigmentation + hypopigmentation 3** |
|  | **QSND** |  |  |  |  | **1.61 ± 4.39** | **Hyperpigmentation 6** |
| **2016 Chung JY^[77]^** | **IPL + tTA** | **15** | **41.38 ± 4.37** |  | **24** | **5.39 ± 5.08** | **No** |
|  | **IPL** |  |  |  |  | **1.47 ± 6.49** | **No** |
| 2016 Lajevardi V^[78]^ | oTA + HQ | 45 | 35.4 ± 5.7 |  | 24 | 7.4 ± 5.8 | Abdominal pain 3, flank pain and edema 1, nausea-vomiting-headache 1 |
|  | HQ | 43 | 37.3 ± 5.8 |  |  | 3.5 ± 3.9 |  |
| **2015 Vachiramon V^[79]^** | **QSND + GA peel** | **15** | **45 (28–69)** | **Ⅲ4, IV9, V2** | **8** | **7.08 ± 2.08** | **Burning 7, erythema 5, hyperpigmentation 2, hypopigmentation 1** |
|  | QSND |  |  |  |  | -0.67 ± 2.01 | Burning 5, erythema 3, hyperpigmentation 2, hypopigmentation 1 |
| 2015 Ibrahim ZA^[80]^ | HQ | 20 |  |  | 12 | 6.67 ± 5.05 | Pruritus 8, erythema 4 |
|  | HQ + GA peel | 20 |  |  |  | 3.97 ± 3.94 | Erythema 6, erosion 4, scaling 2, crustation 8 |
|  | HQ + HA peel | 20 |  |  |  | 7.52 ± 3.93 | Pruritus 4 |
|  | HQ + GA peel + HA peel | 20 |  |  |  | 9.14 ± 4.81 | Pruritus 2, erythema 6, scaling 4, crustation 2 |
|  | Placebo | 20 |  |  |  | 0.00 ± 2.69 |  |
| 2015 Elfar NN^[81]^ | tTA | 20 | 37.0 ± 4.80 | Epidermal 30; dermal 13; mixed 17 | 12 | 1.86 ± 2.21 | Burning pain 20, wheal 20, erythema 5 |
|  | GA peel | 20 | 36.45 ± 6.28 |  |  | 4.06 ± 3.10 | PIH 6 |
| **2015 Banihashemi M^[82]^** | **tTA** | **30** | **25–47** |  | **16** | **7.94 ± 2.62** |  |
|  | **HQ** |  |  |  |  | **7.00 ± 2.25** | **Skin irritation 3** |
| 2015 Padhi T^[83]^ | TCC | 20 |  |  | 8 | 8.43 ± 5.59 | Erythema 3, burning 2 |
|  | TCC + oTA | 20 |  |  |  | 16.05 ± 2.99 | Erythema 2, burning 2, PIH 2, oligomenorrhoea 1 |
| 2014 Lee DB^[84]^ | QSND + placebo | 26 | 42.08 ± 6.56 |  | 20 | 2.46 ± 3.55 | Peel:burning 4; QSND: pain and erythema |
|  | QSND + Jessner peel | 26 | 40.85 ± 7.48 |  |  | 2.93 ± 3.31 |  |
| 2014 Yun WJ^[85]^ | QSND + IPL | 12 | 42.6 ± 1.9 |  | 18 | 6.25 ± 4.09 | Burn 1, dry 3 |
|  | IPL | 12 | 43.4 ± 2.0 |  |  | 2.83 ± 3.12 |  |
| 2014 Mendoza CG^[86]^ | HQ | 15 | 29.04 ± 7.8 | Epidermal 11; mixed 4 | 8 | 0.55 ± 0.62 |  |
|  | Placebo | 15 |  | Epidermal 15 |  | 0.09 ± 0.12 |  |
| **2014 Truchuelo MT^[87]^** | **Tretinoin** | **30** | **39** | **Ⅱ18, Ⅲ12** | **12** | **7.00 ± 4.04** |  |
|  | **Placebo** |  |  |  |  | **5.20 ± 4.41** |  |
| **2014 Jalaly NY^[88]^** | **AFL-CO_2_** | **40** | **42.5 ± 7** | **II21, III16, IV3** | **8** | **6.30 ± 8.91** | **Erythema, burning, edema, scaling** |
|  | **QSND** |  |  |  |  | **8.85 ± 8.10** | **Erythema, edema** |
| **2014 Vachiramon V^[89]^** | **QSND** | **20** | **47.5 ± 6.9** |  | **20** | **8.15 ± 7.62** | **Erythema, stinging** |
|  | **QSND+IPL** |  |  |  |  | **2.30 ± 6.81** | **Erythema, stinging, microcrust 14, hypomelanosis 1** |
| 2013 Shin JU^[90]^ | QSND + oTA | 23 | 44.4 ± 7.9 | III or IV | 12 | 2.90 ± 3.55 | Erythema 23, medication-associated heartburn 2, nausea 1 |
|  | QSND | 21 | 43.2 ± 6.91 |  |  | 1.90 ± 3.60 | Erythema 21 |
| **2013 Kim HS^[91]^** | **QSND** | **26** | **45 (33–64)** | **III or V** | **32** | **2.55 ± 1.36** |  |
|  | **QSND + NAFL** |  |  |  |  | **2.58 ± 1.22** | **Erythema, burning, edema, PIH 2** |
| 2013 Deo KS^[92]^ | KA | 20 |  |  | 12 | 5.57 ± 6.70 |  |
|  | KA + HQ | 20 |  |  |  | 6.29 ± 4.34 |  |
|  | KA + betamethasone valerate | 20 |  |  |  | 3.44 ± 6.99 |  |
|  | KA + HQ + betamethasone valerate | 20 |  |  |  | 8.49 ± 8.21 |  |
| 2013 Chaudhary S^[93]^ | Topical regimen + GA | 20 | 34 (21–40) |  | 24 | 12.40 ± 2.94 | Erythema 4, PIH 3, hypertrichosis 1, burning and stinging 9 |
|  | Topical regimen | 20 |  |  |  | 7.27 ± 3.05 |  |
| 2012 Kar HK^[94]^ | Low-fluence QSND | 21 | 34.05 ± 6.79 | IV20, V40 | 12 | 6.49 ± 6.44 | Hypopigmentation 1 |
|  | GA peel | 19 |  |  |  | 4.35 ± 5.59 | Erythema/Burning 4, PIH 1 |
|  | High-fluence QSND | 20 |  |  |  | 2.19 ± 4.72 | Erythema/Burning 4, hypopigmentation 5, PIH 6 |
| 2012 Figueiredo SL^[95]^ | IPL | 31 | 43.8 ± 7.2 | Ⅱ3, Ⅲ11, Ⅳ13, Ⅴ4 | 24 | 8.70 ± 6.65 | Erythema, pain, PIH 3 |
|  | Placebo | 31 | 45.1 ± 4.3 | Ⅱ4, Ⅲ10, Ⅳ13, Ⅴ4 |  | 0.39 ± 5.28 |  |
| 2012 Bansal C^[96]^ | QSND | 20 | 37.7 ± 6.63 | Ⅲ1, Ⅳ31, Ⅴ28 | 12 | 6.22 ± 4.79 |  |
|  | AA | 20 |  |  |  | 11.00 ± 6.04 | Burning/Stinging 1 |
|  | QSND + AA | 20 |  |  |  | 13.79 ± 6.84 | Erythema 1, burning/stinging 1 |
| 2012 Karn D^[97]^ | oTA | 130 | 30.3 ± 9.01 | Epidermal 93; dermal 14; mixed 23 | 12 | 3.24 ± 2.70 | Oligomenorrhea 16, belching 12, abdominal cramps 9 |
|  | Placebo | 130 |  | Epidermal 80; dermal 14; mixed 36 |  | 2.34 ± 3.21 |  |
| **2012 Sobhi RM^[98]^** | **GA peel** | **14** | **39.36 ± 7.397** | **Ⅳ9, Ⅴ5** | **4** | **1.13 ± 2.68** | **PIH 2, dryness 1, burning 1, blister 4** |
|  | **tVC** |  |  |  |  | **1.18 ± 2.85** | **Burning 1, electric shock 1** |
| **2012 Hong SP^[99]^** | **NAFL** | **17** | **35.4 ± 4.67** | **Ⅲ or Ⅳ** | **12** | **1.75 ± 2.63** | **erythema 3, PIH 5** |
|  | **TCA peel** |  |  |  |  | **3.53 ± 2.80** | **erythema 4, PIH 5** |
| 2011 Kroon MW^[100]^ | NAFL | 10 | 35.3 | Ⅱ2, Ⅲ4, Ⅳ3, Ⅴ1 | 12 | -0.19 ± 4.83 | Erythema 75%, burning 58% |
|  | TCC | 10 | 42 | Ⅱ3, Ⅲ4, Ⅳ2, Ⅴ1 |  | 1.30 ± 6.81 | Erythema 25%, burning 20%, scaling 55% |
| **2011 Park KY^[12]^** | **QSND + GA peel** | **16** | **43.94 ± 8.64** | **Ⅲ or Ⅳ** | **5** | **5.80 ± 1.60** | **Erythema, burning, edema** |
|  | **QSND** |  |  |  |  | **3.50 ± 1.85** |  |
| 2011 Farshi S^[101]^ | HQ | 15 | 34.6 ± 6.6 |  | 8 | 1.00 ± 3.41 | Erythema 2, irritation 7, pruritus 1 |
|  | AA | 14 |  |  |  | 3.80 ± 3.20 | Irritation 2 |
| **2011 Faghihi G^[102]^** | **GA peel** | **63** | **37.3 ± 9.13** |  | **12** | **2.40 ± 3.27** | **PIH 3** |
|  | **Tretinoin** |  |  |  |  | **2.70 ± 3.43** | **PIH 1** |
| 2010 Trelles MA^[103]^ | TCC | 10 | 35.6 ± 6.3 | Ⅱ10, Ⅲ12, Ⅳ8 | 4 | 18.10 ± 6.84 |  |
|  | AFL-CO_2_ | 10 |  |  |  | 15.30 ± 6.26 | Stinging/Burning |
|  | AFL-CO_2_ + TCC | 10 |  |  |  | 20.30 ± 6.71 | Stinging/Burning |
| **2010 Wattanakrai P^[104]^** | **QSND + HQ** | **22** |  | **Ⅲ10, Ⅳ6, Ⅴ6** | **7** | **16.60 ± 1.56** | **Erythema, burning, edema, hypopigmentation 3** |
|  | **HQ** |  |  |  |  | **5.30 ± 1.63** |  |
| **2009 Safoury OS^[105]^** | **TCA peel** | **20** | **38.25** | **Ⅲ or Ⅳ** | **10** | **2.19 ± 1.49** | **Swelling 2, erythema 5, discomfort 4, PIH 2, folliculitis/acne 5** |
|  | **TCA peel + Jessner peel** |  |  |  |  | **2.68 ± 1.34** | **Swelling 2, erythema 5, discomfort 16, folliculitis/acne 5** |
| 2008 Garg VK^[106]^ | GA peel | 15 | 31.54 (17–45) | Ⅳ 28.6%，Ⅴ 71.4% | 12 | 3.76 ± 3.22 | Hyperpigmentation 20%, milia 26.6%, nodulocystic acne 5.5% |
|  | Tretinoin + GA peel | 17 |  |  |  | 4.28 ± 7.44 | Hyperpigmentation 14.3%, erythema and desquamation 53% |
|  | HQ + GA peel | 18 |  |  |  | 5.72 ± 6.72 | Hyperpigmentation 5.5%, erythema and desquamation 22%, persistent erythema 5.5% |
| 2002 Sarkar R^[107]^ | GA peel + TCC | 20 | 31.45 (19–44) |  | 21 | 15.19 ± 5.88 | Erythema 20, PIH 2 |
|  | TCC | 20 | 31.05 (21–45) |  |  | 11.88 ± 4.70 | Erythema 8 |
| 1994 Candance K^[108]^ | Tretinoin | 15 | 53 (44–67) | Epidermal 8; dermal 4; mixed 3 | 40 | 4.80 ± 7.33 | Erythema/Peeling 10 |
|  | Placebo | 13 | 52 (22–70) | Epidermal 5; dermal 7; mixed 3 |  | 1.60 ± 9.19 | Erythema/Peeling 1 |

*25 Studies highlighted in boldface were split-face control studies.

**Abbreviation:** AA, Azelaic acid; AFL-CO_2_, ablative fractional laser-CO_2_ laser; AFL-Er:YAG, ablative fractional laser-Er:YAG laser; FbTc, fluocinolone-based combination cream; GA, glycolic acid; HA, hyaluronic acid; HQ, hydroquinone; IPL, intense pulsed light; KA, kojic acid; MN, microneedling; N, number of patients with treatment; NAFL, non-ablative fractional laser; oTA, oral tranexamic acid; PICO, picosecond laser; QSND, q-switch Nd:Yag 1064-nm laser; SA, salicylic acid; TCA, trichloroacetic acid; TCC, triple combination cream; tTA, topical tranexamic acid; tVC, topical vitamin C; PIH: post-inflammatory hyperpigmentation.

1. Minni K, Poojary S. Efficacy and safety of oral tranexamic acid as an adjuvant in Indian patients with melasma: a prospective, interventional, single-centre, triple-blind, randomized, placebo-control, parallel group study. J Eur Acad Dermatol Venereol. 2020;34(11), 2636-2644.
2. Kaur A, Bhalla M, Pal TG, Sandhu J. Clinical Efficacy of Topical Tranexamic Acid With Microneedling in Melasma. Dermatol Surg. 2020;46(11), e96-e101.
3. Cassiano D, Esposito ACC, Hassun K, Bagatin E, Lima MMDA. Efficacy and safety of microneedling and oral tranexamic acid in the treatment of facial melasma in women: An open, evaluator-blinded, randomized clinical trial. J Am Acad Dermatol. 2020;83(4), 1176-1178.
4. Shihab N, Prihartono J, Tovar-Garza A, Agustin T, Legiawati L. Randomised, controlled, double-blind study of combination therapy of oral tranexamic acid and topical hydroquinone in the treatment of melasma. Australas J Dermatol. 2020;61(3), 237-242.
5. Kaleem S, Ghafoor R, Khan S. Comparison of efficacy of Tranexamic Acid Mesotherapy versus 0.9% normal Saline for Melasma; A split face study in a Tertiary Care Hospital of Karachi. Pak J Med Sci. 2020;36(5), 930-934.
6. Otb S, Shaarawy E, Sadek A, Abdulla N, Agamia N, Soliman M, et al. A Split Face Comparative Study between Intradermal Tranexamic Acid and Erbium-YAG laser in Treatment of Melasma. J Dermatolog Treat. 2020;undefined(undefined), 1-22.
7. Shamsi MS, Mozayyeni A, Shamsi MM, Aflatoonian M. Efficacy of microneedling plus topical 4% tranexamic acid solution vs 4% hydroquinone in the treatment of melasma: A single-blind randomized clinical trial. J Cosmet Dermatol. 2020;19(11), 2906-2911.
8. Mekawy KMM, Sadek A, Seddeik Abdel-Hameed AK. Micro-needling versus fractional carbon dioxide laser for delivery of tranexamic acid in the treatment of melasma: A split-face study. J Cosmet Dermatol. 2021;20(2), 460-465.
9. Garg S, Vashisht KR, Makadia S. A prospective randomized comparative study on 60 Indian patients of melasma, comparing pixel Q-switched NdYAG (1064nm), super skin rejuvenation (540nm) and ablative pixel erbium YAG (2940nm) lasers, with a review of the literature. J Cosmet Laser Ther. 2019;21(5), 297-307.
10. Saka S, Raghu RRG, Babu KR, Kotha S, Tatavarthi R. Efficacy of topical tranexamic acid in melasma-A randomized placebo-controlled split face study. IJPSR. 2019;10(5): 2583-2586.
11. Janney MS, Subramaniyan R, Dabas R, Lal S, Das NM, Godara SK. A Randomized Controlled Study Comparing the Efficacy of Topical 5% Tranexamic Acid Solution versus 3% Hydroquinone Cream in Melasma. J Cutan Aesthet Surg. 2019;12(1), 63-67.
12. Zhao H, Li M, Zhang X, Li L, Yan Y, Wang B. Comparing the efficacy of Myjet-assisted tranexamic acid and vitamin C in treating melasma: A split-face controlled trial. J Cosmet Dermatol. 2020;19(1), 47-54.
13. Tehranchinia Z, Saghi B, Rahimi H. Evaluation of Therapeutic Efficacy and Safety of Tranexamic Acid Local Infiltration in Combination with Topical 4% Hydroquinone Cream Compared to Topical 4% Hydroquinone Cream Alone in Patients with Melasma: A Split-Face Study. Dermatol Res Pract. 2018;(undefined), 8350317.
14. Chalermchai T, Rummaneethorn P. Effects of a fractional picosecond 1,064 nm laser for the treatment of dermal and mixed type melasma. J Cosmet Laser Ther. 2018;20(3), 134-139.
15. Colferai MMT, Miquelin GM, Steiner D. Evaluation of oral tranexamic acid in the treatment of melasma. J Cosmet Dermatol. 2018;undefined(undefined), undefined.
16. Vanaman Wilson MJ, Jones IT, Bolton J, Larsen L, Fabi SG. The Safety and Efficacy of Treatment With a 1,927-nm Diode Laser With and Without Topical Hydroquinone for Facial Hyperpigmentation and Melasma in Darker Skin Types. Dermatol Surg. 2018;44(10), 1304-1310.
17. Tawfic SO, Abdel Halim DM, Albarbary A, Abdelhady M. Assessment of combined fractional CO and tranexamic acid in melasma treatment. Lasers Surg Med. 2019;51(1), 27-33.
18. Atefi N, Dalvand B, Ghassemi M, Mehran G, Heydarian A. Therapeutic Effects of Topical Tranexamic Acid in Comparison with Hydroquinone in Treatment of Women with Melasma. Dermatol Ther (Heidelb). 2017;7(3), 417-424.
19. Choi YJ, Nam JH, Kim JY, Min JH, Park KY, Ko EJ, et al. Efficacy and safety of a novel picosecond laser using combination of 1 064 and 595 nm on patients with melasma: A prospective, randomized, multicenter, split-face, 2% hydroquinone cream-controlled clinical trial. Lasers Surg Med. 2017;49(10), 899-907.
20. Dayal S, Sahu P, Dua R. Combination of glycolic acid peeling and topical 20% azelaic acid cream in melasma patients: efficacy and improvement in quality of life. J Cosmet Dermatol. 2017;16(1), 35-42.
21. Lu J, Yang L, Xu P, Bian F, Zhang H. Whitening Efficacy of Tranexamic Acid Cataplasm on Melasma in Chinese Women. Integr Med Int. 2017;4:154–160.
22. Abdel-Meguid AM, Taha EA, Ismail SA. Combined Jessner Solution and Trichloroacetic Acid Versus Trichloroacetic Acid Alone in the Treatment of Melasma in Dark-Skinned Patients. Dermatol Surg. 2017;43(5), 651-656.
23. Balevi A, Ustuner P, Özdemir M. Salicylic acid peelinging combined with vitamin C mesotherapy versus salicylic acid peelinging alone in the treatment of mixed type melasma: A comparative study. J Cosmet Laser Ther. 2017;19(5), 294-299.
24. Ustuner P, Balevi A, Ozdemir M. A split-face, investigator-blinded comparative study on the efficacy and safety of Q-switched Nd:YAG laser plus microneedling with vitamin C versus Q-switched Nd:YAG laser for the treatment of recalcitrant melasma. J Cosmet Laser Ther. 2017;19(7), 383-390.
25. Chung JY, Lee JH, Lee JH. Topical tranexamic acid as an adjuvant treatment in melasma: Side-by-side comparison clinical study. J Dermatolog Treat. 2016;27(4), 373-7.
26. Lajevardi V, Ghayoumi A, Abedini R, Hosseini H, Goodarzi A, Akbari Z, et al. Comparison of the therapeutic efficacy and safety of combined oral tranexamic acid and topical hydroquinone 4% treatment vs. topical hydroquinone 4% alone in melasma: a parallel-group, assessor- and analyst-blinded, randomized controlled trial with a short-term follow-up. J Cosmet Dermatol. 2017;16(2), 235-242.
27. Vachiramon V, Sahawatwong S, Sirithanabadeekul P. Treatment of melasma in men with low-fluence Q-switched neodymium-doped yttrium-aluminum-garnet laser versus combined laser and glycolic acid peelinging. Dermatol Surg. 2015;41(4), 457-65.
28. Ibrahim ZA, Gheida SF, El Maghraby GM, Farag ZE. Evaluation of the efficacy and safety of combinations of hydroquinone, glycolic acid, and hyaluronic acid in the treatment of melasma. J Cosmet Dermatol. 2015;14(2), 113-23.
29. Elfar NN, El-Maghraby GM. Efficacy of Intradermal Injection of Tranexamic Acid, Topical Silymarin and Glycolic Acid Peelinging in Treatment of Melasma: A Comparative Study. J Clin Exp Dermatol Res. 2015;6:3.
30. Banihashemi M, Zabolinejad N, Jaafari Mahmoud R, Salehi M, Jabari A. Comparison of therapeutic effects of liposomal Tranexamic Acid and conventional Hydroquinone on melasma. J Cosmet Dermatol. 2015;14(3), 174-7.
31. Padhi T, Pradhan S. Oral Tranexamic Acid with Fluocinolone-Based Triple Combination Cream Versus Fluocinolone-Based Triple Combination Cream Alone in Melasma: An Open Labeled Randomized Comparative Trial. Indian J Dermatol. 2015;60(5), 520.
32. Lee DB, Suh HS, Choi YS. A comparative study of low-fluence 1064-nm Q-switched Nd:YAG laser with or without chemical peelinging using Jessner's solution in melasma patients. J Dermatolog Treat. 2014;25(6), 523-8.
33. Yun WJ, Moon HR, Lee MW, Choi JH, Chang SE. Combination treatment of low-fluence 1,064-nm Q-switched Nd: YAG laser with novel intense pulse light in Korean melasma patients: a prospective, randomized, controlled trial. Dermatol Surg. 2014;40(8), 842-50
34. Mendoza CG, Singzon IA, Handog EB. A randomized, double-blind, placebo-controlled clinical trial on the efficacy and safety of 3% Rumex occidentalis cream versus 4% hydroquinone cream in the treatment of melasma among Filipinos. Int J Dermatol. 2014;53(11), 1412-6.
35. Truchuelo MT, Jiménez N, Jaén P. Assessment of the efficacy and tolerance of a new combination of retinoids and depigmenting agents in the treatment of melasma. J Cosmet Dermatol. 2014;13(4), 261-8.
36. Jalaly NY, Valizadeh N, Barikbin B, Yousefi M. Low-power fractional CO₂ laser versus low-fluence Q-switch 1,064 nm Nd:YAG laser for treatment of melasma: a randomized, controlled, split-face study. Am J Clin Dermatol. 2014;15(4), 357-63.
37. Vachiramon V, Sirithanabadeekul P, Sahawatwong S. Low-fluence Q-switched Nd: YAG 1064-nm laser and intense pulsed light for the treatment of melasma. J Eur Acad Dermatol Venereol. 2015;29(7), 1339-46.
38. Shin JU, Park J, Oh SH, Lee JH. Oral tranexamic acid enhances the efficacy of low-fluence 1064-nm quality-switched neodymium-doped yttrium aluminum garnet laser treatment for melasma in Koreans: a randomized, prospective trial. Dermatol Surg. 2013;39(null), 435-42.
39. Kim HS, Kim EK, Jung KE, Park YM, Kim HO, Lee JY. A split-face comparison of low-fluence Q-switched Nd: YAG laser plus 1550 nm fractional photothermolysis vs. Q-switched Nd: YAG monotherapy for facial melasma in Asian skin. J Cosmet Laser Ther. 2013;15(3), 143-9.
40. Deo KS, Dash KN, Sharma YK, Virmani NC, Oberai C. Kojic Acid vis-a-vis its Combinations with Hydroquinone and Betamethasone Valerate in Melasma: A Randomized, Single Blind, Comparative Study of Efficacy and Safety. Indian J Dermatol. 2013;58(4), 281-5.
41. Chaudhary S, Dayal S. Efficacy of combination of glycolic acid peelinging with topical regimen in treatment of melasma. J Drugs Dermatol. 2013;12(10), 1149-53.
42. Kar HK, Gupta L, Chauhan A. A comparative study on efficacy of high and low fluence Q-switched Nd:YAG laser and glycolic acid peeling in melasma. Indian J Dermatol Venereol Leprol. 2012;78(2), 165-71.
43. Figueiredo SL, Trancoso SS. Single-session intense pulsed light combined with stable fixed-dose triple combination topical therapy for the treatment of refractory melasma. Dermatol Ther. 2012;25(5), 477-80.
44. Bansal C, Naik H, Kar HK, Chauhan A. A Comparison of Low-Fluence 1064-nm Q-Switched Nd: YAG Laser with Topical 20% Azelaic Acid Cream and their Combination in Melasma in Indian Patients. J Cutan Aesthet Surg. 2012;5(4), 266-72.
45. Karn D, Kc S, Amatya A, Razouria EA, Timalsina M. Oral tranexamic acid for the treatment of melasma. Kathmandu Univ Med J (KUMJ). 2012;10(40), 40-3.
46. Sobhi RM, Sobhi AM. A single-blinded comparative study between the use of glycolic acid 70% peeling and the use of topical nanosome vitamin C iontophoresis in the treatment of melasma. J Cosmet Dermatol. 2012;11(1), 65-71.
47. Hong SP, Han SS, Choi SJ, Kim MS, Won CH, Lee MW, et al. Split-face comparative study of 1550 nm fractional photothermolysis and trichloroacetic acid 15% chemical peelinging for facial melasma in Asian skin. J Cosmet Laser Ther. 2012;14(2), 81-6.
48. Kroon MW, Wind BS, Beek JF, van der Veen JP, Nieuweboer-Krobotová L, Bos JD, et al. Nonablative 1550-nm fractional laser therapy versus triple topical therapy for the treatment of melasma: a randomized controlled pilot study. J Am Acad Dermatol. 2011;64(3), 516-23.
49. Farshi S. Comparative study of therapeutic effects of 20% azelaic acid and hydroquinone 4% cream in the treatment of melasma. J Cosmet Dermatol. 2011;0(4), 282-7.
50. Faghihi G, Shahingohar A, Siadat AH. Comparison between 1% tretinoin peelinging versus 70% glycolic acid peelinging in the treatment of female patients with melasma. J Drugs Dermatol. 2011;10(12), 1439-42.
51. Trelles MA, Velez M, Gold MH. The treatment of melasma with topical creams alone, CO2 fractional ablative resurfacing alone, or a combination of the two: a comparative study. J Drugs Dermatol. 2010;9(4), 315-22.
52. Wattanakrai P, Mornchan R, Eimpunth S. Low-fluence Q-switched neodymium-doped yttrium aluminum garnet (1,064 nm) laser for the treatment of facial melasma in Asians. Dermatol Surg. 2010;36(1), 76-87.
53. Safoury OS, Zaki NM, El Nabarawy EA, Farag EA. A study comparing chemical peelinging using modified Jessner's solution and 15% trichloroacetic Acid versus 15% trichloroacetic acid in the treatment of melasma. Indian J Dermatol. 2009;54(1), 41-5.
54. Garg VK, Sarkar R, Agarwal R. Comparative evaluation of beneficiary effects of priming agents (2% hydroquinone and 0.025% retinoic acid) in the treatment of melasma with glycolic acid peelings. Dermatol Surg. 2008;34(8), 1032-9; discussion 1340.
55. Sarkar R, Kaur C, Bhalla M, Kanwar AJ. The combination of glycolic acid peelings with a topical regimen in the treatment of melasma in dark-skinned patients: a comparative study. Dermatol Surg. 2002;28(9), 828-32; discussion 832.
56. Kimbrough-Green CK, Griffiths CE, Finkel LJ, Hamilton TA, Bulengo-Ransby SM, Ellis CN, Voorhees JJ. Topical retinoic acid (tretinoin) for melasma in black patients. A vehicle-controlled clinical trial. Arch Dermatol. 1994;130(6), 727-33.
